# Supplementary figures and images for: How well do models of visual cortex generalize to out of distribution samples?
Source: PLoS Comput Biol. 2024 May 31;20(5):e1011145. doi: 10.1371/journal.pcbi.1011145 (PMC11216589; doi:10.1371/journal.pcbi.1011145)

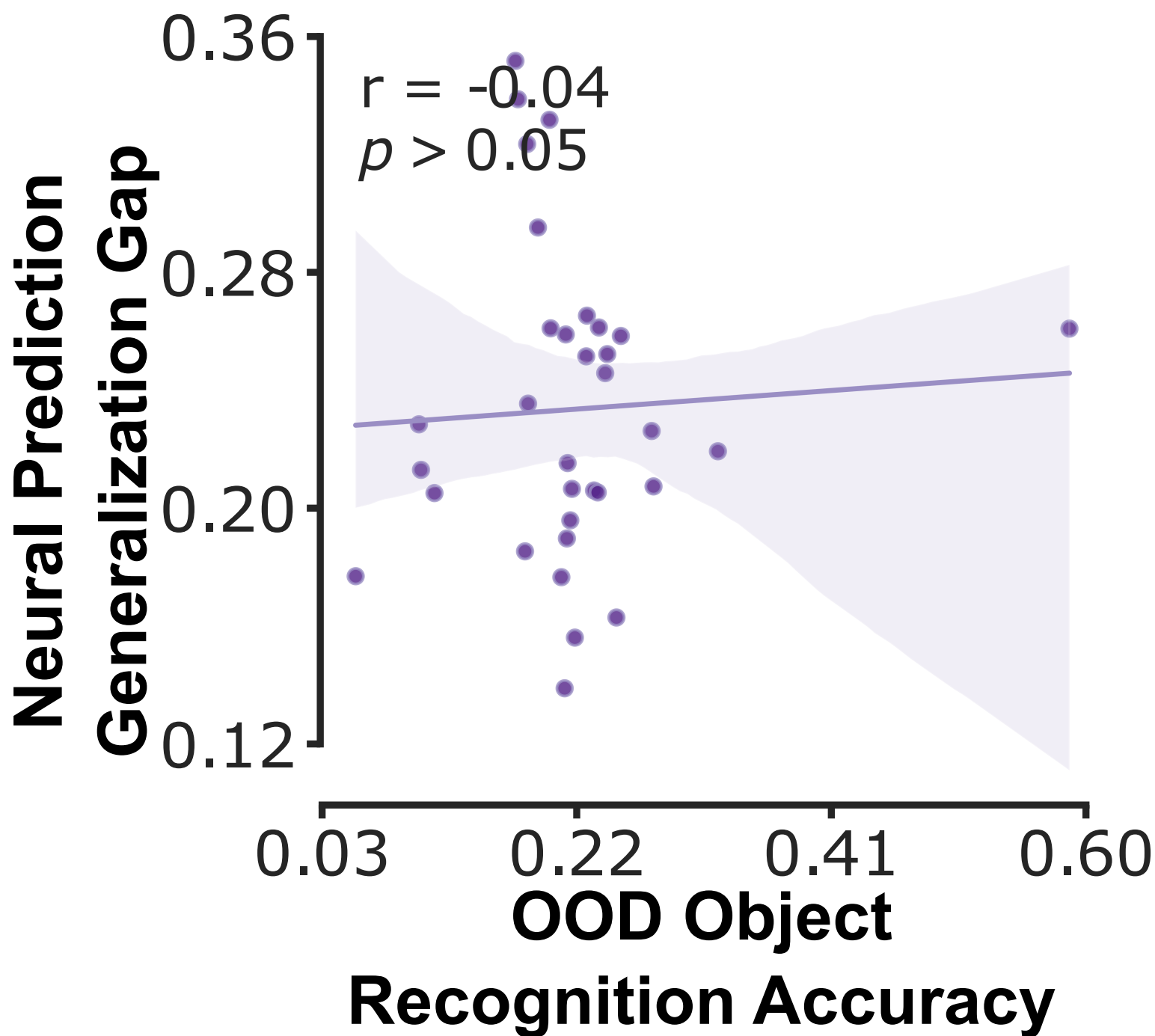

Supplement: S1 Fig — OOD object recognition accuracy for each model is computed as the average accuracy of that model across 5 OOD object recognition benchmarks. Each dot corresponds to one neural network model. Neural prediction generalization gap is computed as the difference between the neural predictivity on the natural and synthetic domains. (PDF) [file pcbi.1011145.s001.pdf]

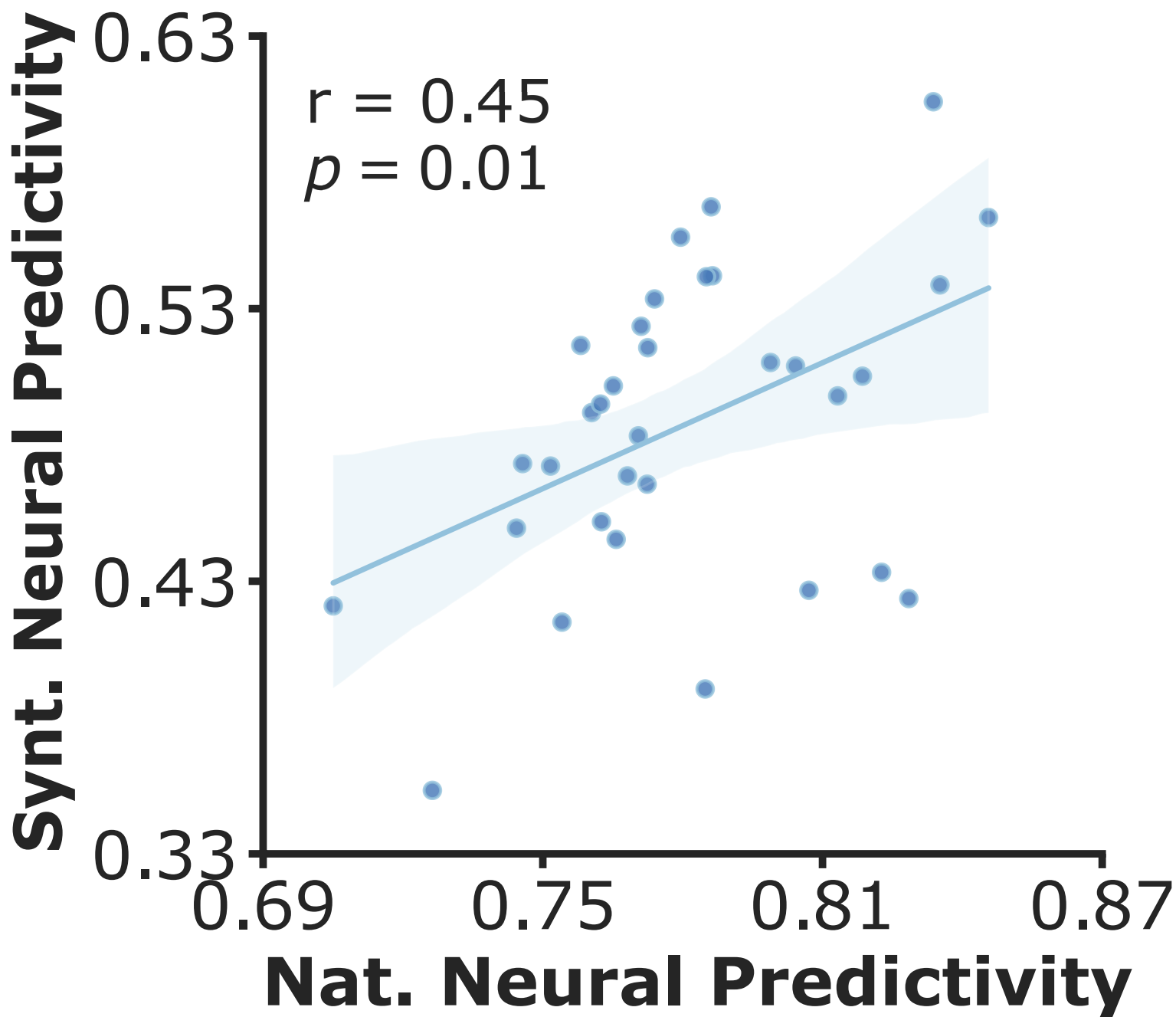

Supplement: S2 Fig — Significant correlation exists between the neural predictivity in natural and synthetic domains. Each dot corresponds to one neural network model. (PDF) [file pcbi.1011145.s002.pdf]

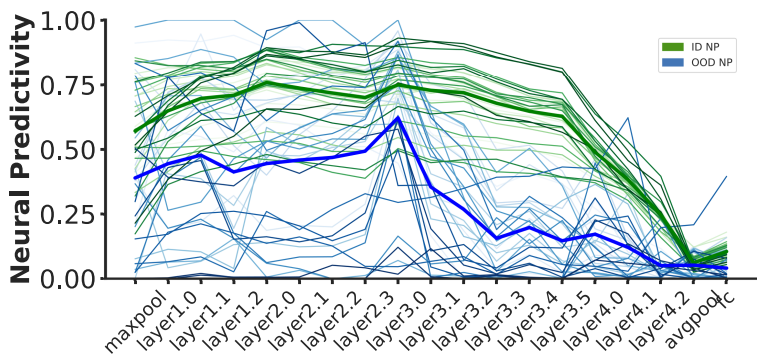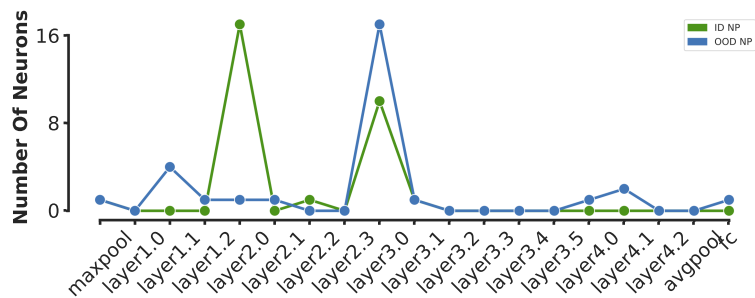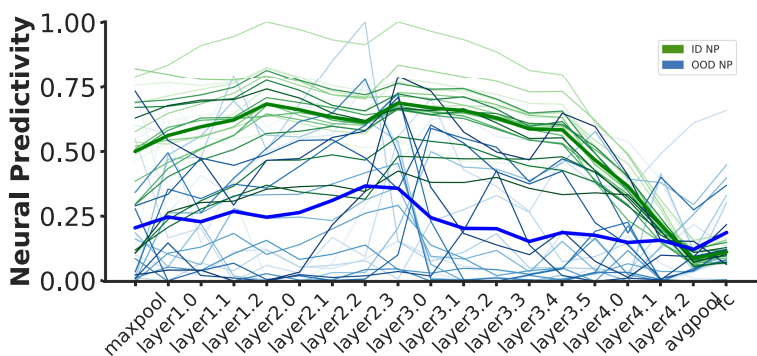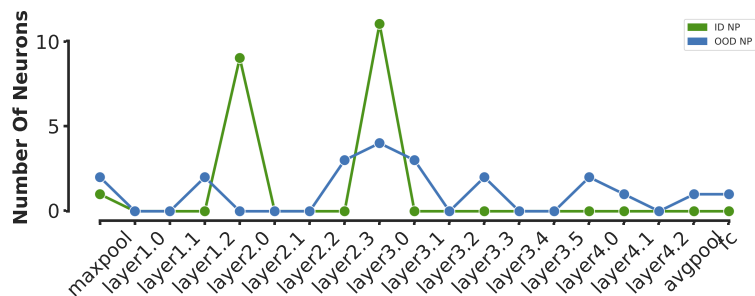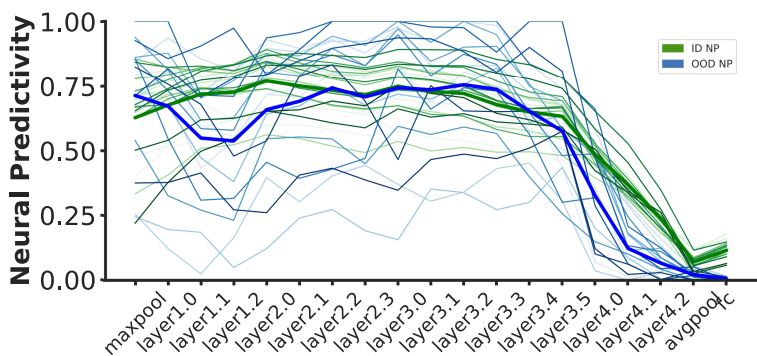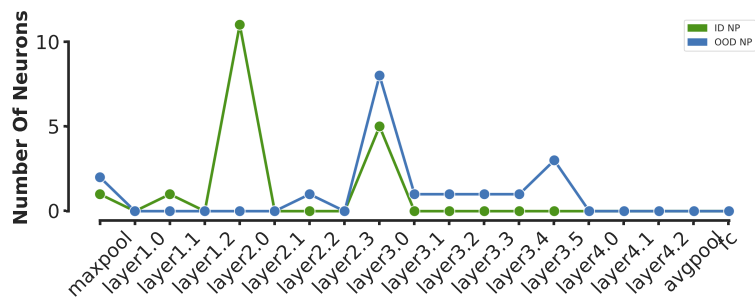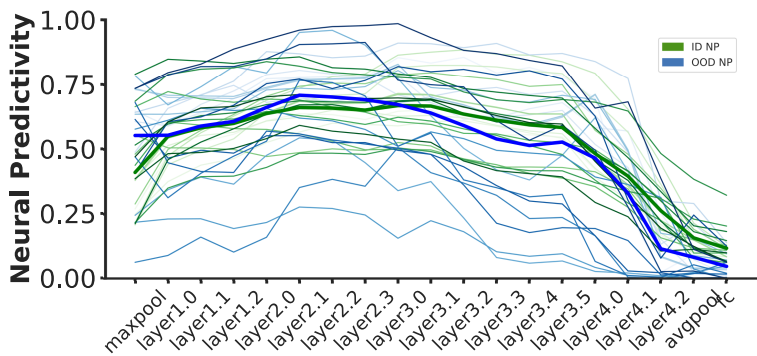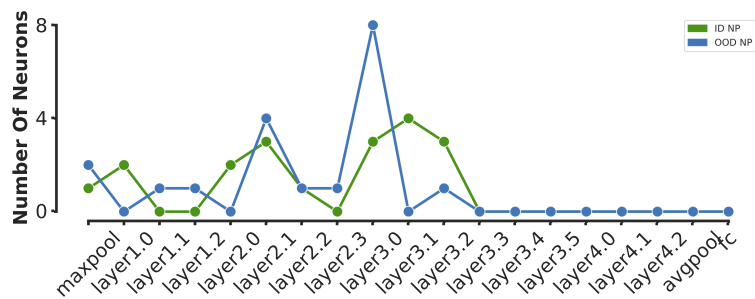

Supplement: S3 Fig — (left column) Neural predictivity scores from unit activity in each layer of ResNet50 architecture for individual neuronal sites recorded during different sessions and different animal subjects. From top, rows correspond to M-S2, M-S3,M-S4, and S-S2. Colors correspond to the neural predictivity score on natural (green) and synthetic (blue) domains. Different shades correspond to different neuronal site in the same animal. Bold lines correspond to the average predictivity score in each domain across all neuronal sites within that animal’s session; (right column) Number of neurons with highest neural predictivity in a given layer corresponding to the same subplot in a. Colors are the same as those in the left column. (PDF) [file pcbi.1011145.s003.pdf]

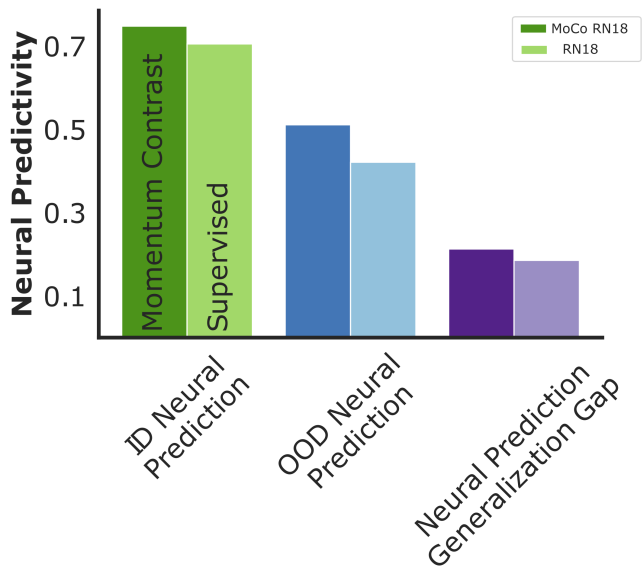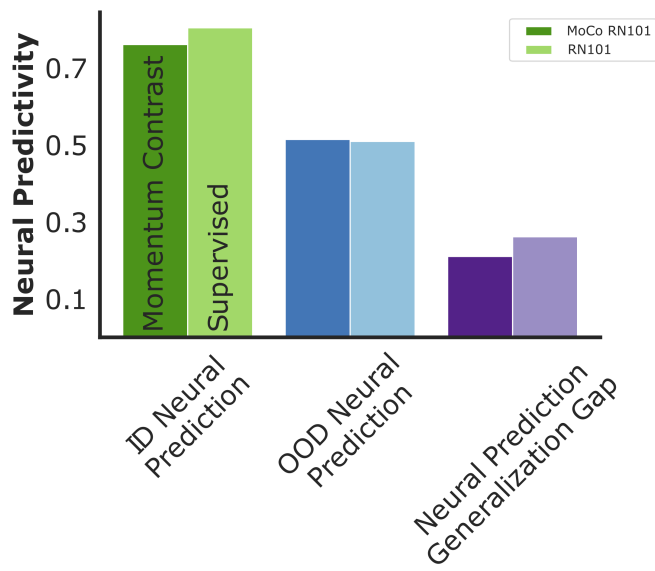

Supplement: S4 Fig — Comparison of ID (green), OOD (blue), and generalization gap (purple) in neural predictivity on ResNet18 (left) and ResNet101 (right) variations of the ResNet architecture. MoCo improves OOD neural predictivity on both architectures. (PDF) [file pcbi.1011145.s004.pdf]

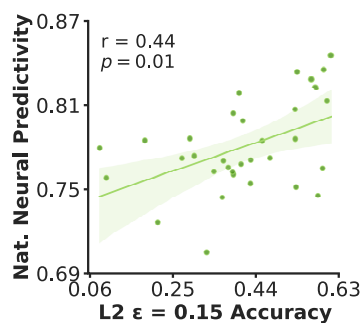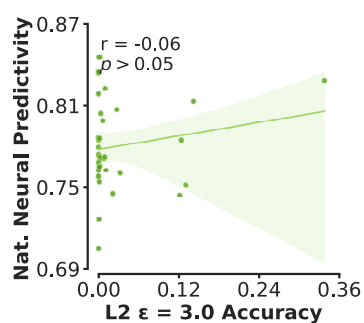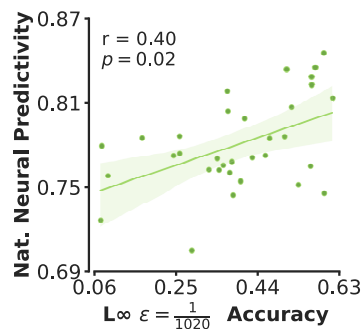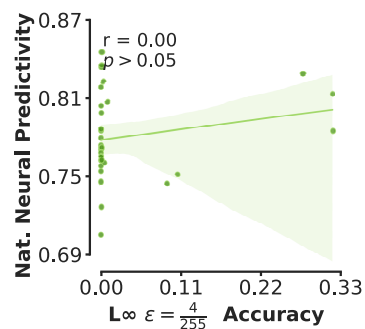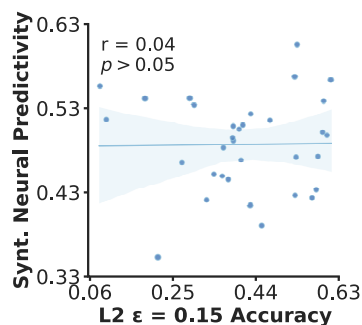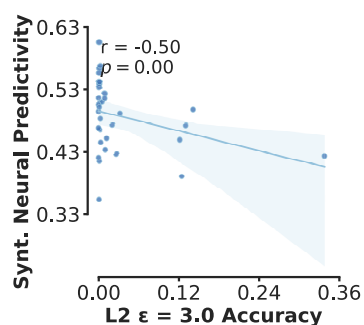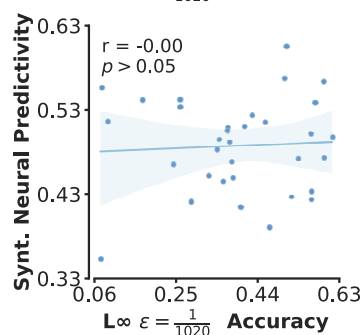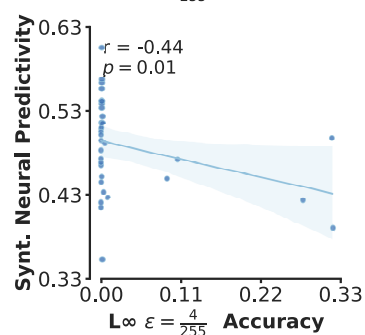

Supplement: S5 Fig — Top and bottom rows illustrate the scatter plots for natural and synthetic domains respectively. Each column corresponds to one measure of robustness used to compute robust accuracy. Neural predictivity is significantly correlated with robustness only for small values of ε for each norm. (PDF) [file pcbi.1011145.s005.pdf]

**A**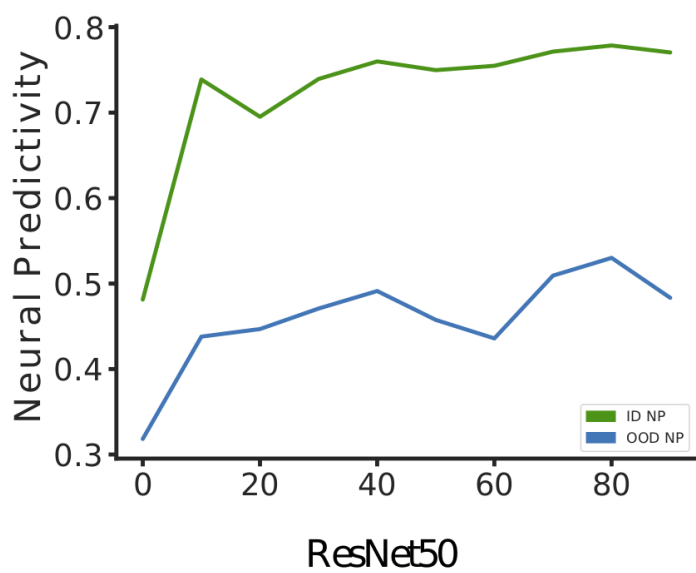**B**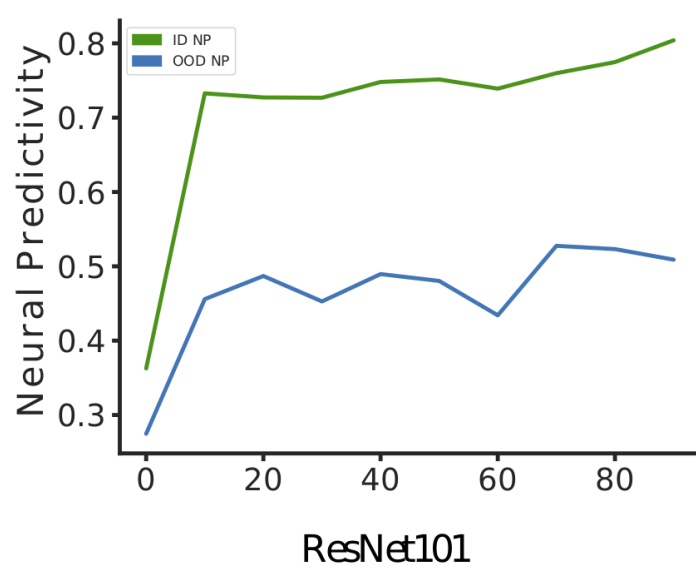

Supplement: S6 Fig — Neural predictivity in both natural and synthetic domains increases during training of ResNet50 and ResNet101 neural networks. (PDF) [file pcbi.1011145.s006.pdf]

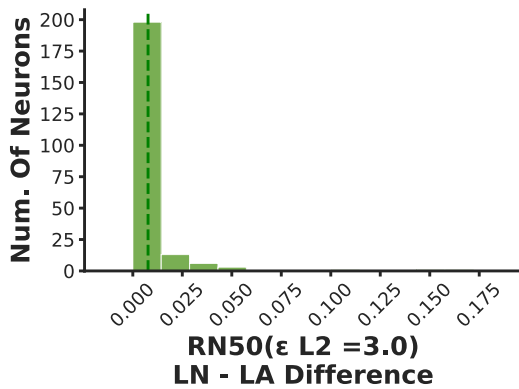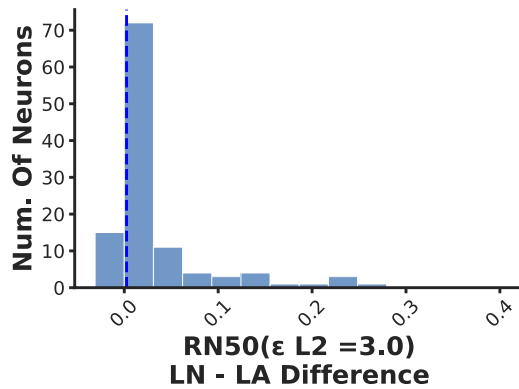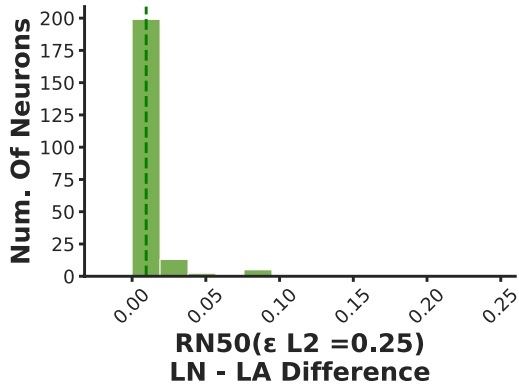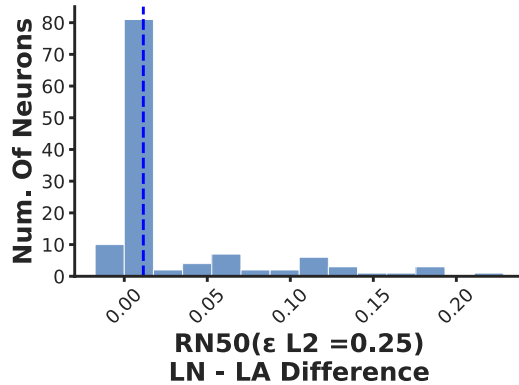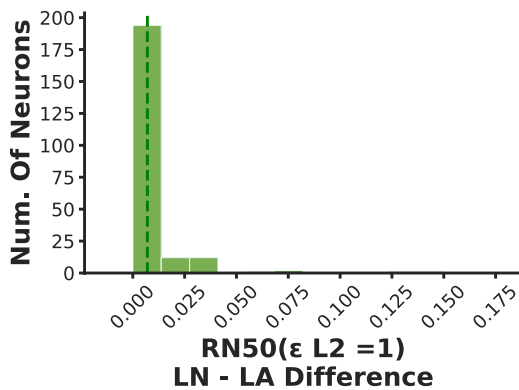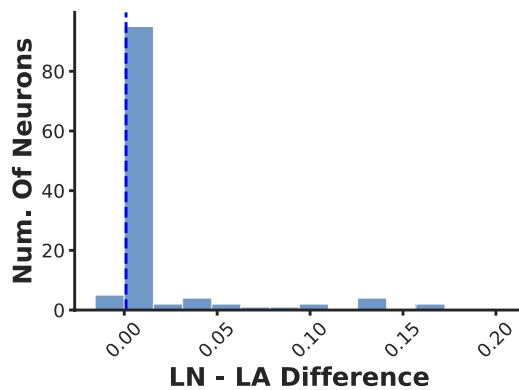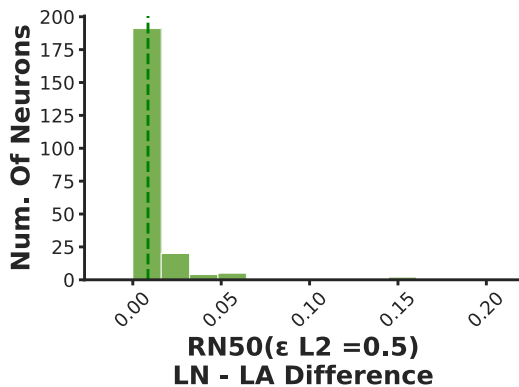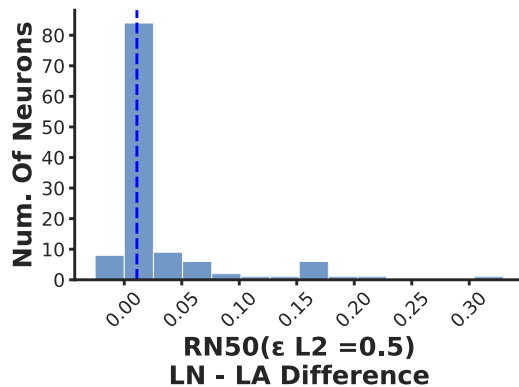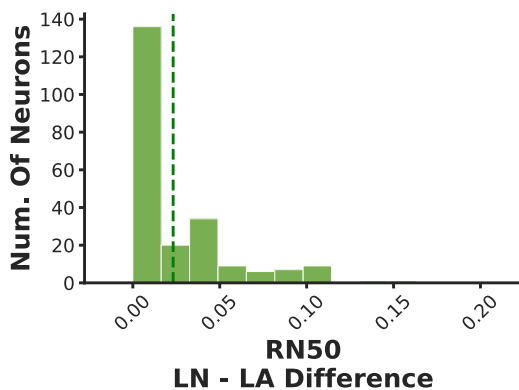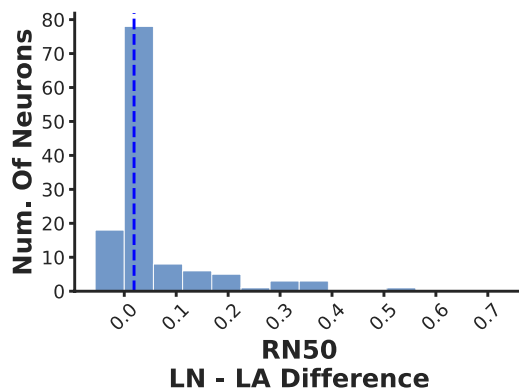

Supplement: S7 Fig — The histograms shows the distribution of difference of ID (left column; green) and OOD(right column; blue) neural predictivity between Layer-Neuron and Layer-Area mapping approach for each neuronal site. The models includes those in Fig 7B. The dashed vertical lines denote the mean of the distribution. A consistent positive mean value illustrates that more than a half of the neurons get their prediction performance improved by using the Layer-Neuron approach. (PDF) [file pcbi.1011145.s007.pdf]

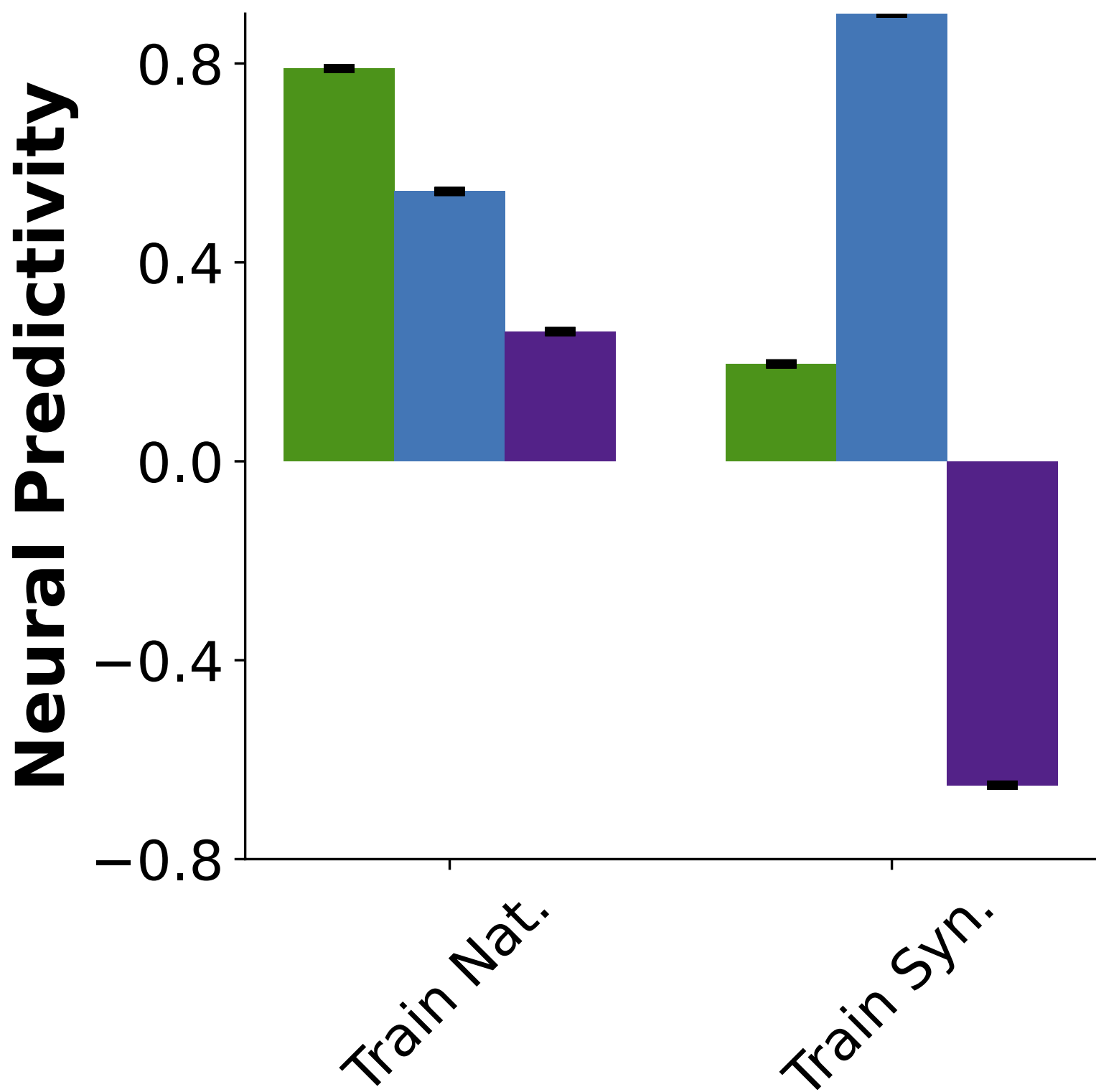

Supplement: S8 Fig — Comparison of Nat. and Syn. predictivity scores for ResNet50 model when the regression model was fitted on naturalistic data (left) and synthetic data (right) without using PCA. The regression model fitted to the synthetic domain shows worse generalization to the naturalistic domain similar to Fig 2E. (PDF) [file pcbi.1011145.s008.pdf]
