# Supplementary material for: How well do models of visual cortex generalize to out of distribution samples?
Source: PLoS Comput Biol. 2024 May 31;20(5):e1011145. doi: 10.1371/journal.pcbi.1011145 (PMC11216589; doi:10.1371/journal.pcbi.1011145)
Supplement: Attachment — Submitted filename: response_letter.pdf [file pcbi.1011145.s009.pdf]

We would like to sincerely thank the reviewers for taking the time to review our manuscript and to provide thoughtful comments and feedback. The reviewers raised several concerns about the clarity and structure of the manuscript (R1 and R2), the significance of the proposed Layer-Neuron approach for making predictive mappings (R1 and R2), and the appropriateness of using naturally-tuned PCA (R2). To address these concerns, we ran new experiments and analyses, revised Figures 2 and 3, added two additional supplementary figures (Figures S7 and S8), and substantially revised the main text. We believe that these changes have significantly improved the quality and clarity of our manuscript and addressed all of the reviewers concerns. We remain open to discussing any further questions or concerns.

Below, we respond to each of the reviewer's comments in detail.

Reviewer #1

"Figure 2 caption. The caption does not describe subfigure d and e
Line 146: There is no Fig 1e. I assume the authors are referring to Fig 2e"
We thank the reviewer for bringing this issue to our attention. We updated the figure 2 caption accordingly and added the missing descriptions. Also, fixed the error in referring to Figure 2e.

"Fig 3 b. Why is there only a very small number of neurons explained (now many are there in total?) and why are they clustered to specific layers?"
First, we would like to clarify that each row of plots in Figure 3 a and b shows results from an example session for each animal (Monkey M, N, S). On each of the subplots which correspond to an example session, the number of neurons is equal to the number of neural sites recorded in that session with an internal consistency of greater than 0.7. Across all sessions, the dataset contains 232 neuronal sites. The internal consistency of 224 and 145 neuronal sites passed the threshold of 0.7 on ID and OOD sessions respectively and were considered in our further analyses including in Figure 3 plots. We added this information to the main text at lines 143 - 146. Second, each subplot in Figure 3b shows the number of neurons that are best explained by each model layer. Indeed, as pointed out by the reviewer and also highlighted in our manuscript (lines 205-206), most neurons tended to cluster in one or two of the model layers. This suggests that the unit activity in 2-3 layers of the neural network model were most similar to the neural responses recorded from area V4 of the three monkeys. In other words, neurons in area V4 can functionally be best explained by considering the model's unit activity in those layers. Data for 3 animal-sessions were included in Figure 3 and the rest of the sessions were included in the supplementary Figure S3.

"Fig 3 c. The Distributions in 3b suggest that the histogram of the number of neurons is shifted left in the case of the green line (natural images) compared to the blue line (synthetic images), but Fig3c shows the opposite. Are the histograms in 3b atypical?"

Thank you for pointing out this inconsistency. The layer differences presented in figure 3C are calculated as the [best ID layer - the best OOD layer]. Therefore, a negative layer difference indicates that the best ID layer appears earlier than the best OOD layer which is consistent with the plots in Fig 3b. We found that our description of this plot in the main text was inconsistent with the plot. To address this, we added additional details in Figure 3 caption and revised the main text to clarify this point (lines 210-212, 514-519).

"Line 200: You say that on out-of-distribution samples the Layer-Neruon mapping improved substantially, yet the 0.487 and 0.479 are very close, also given the big variety in the data Fig 3d and a considerable number in Fig 3d also decreases from LA to LN."

We thank the reviewer for their comment. To clarify, on lines [201- 202] in the original manuscript, we wrote "On out-of-distribution samples, the Layer-Neuron mapping assumption substantially improved the predictivity scores for **many** of the models, however across **all** models the improvement **was not statistically significant**". We understand that this statement may have been obscure and slightly confusing. Therefore, we revised the text to clarify this point further [lines 223-224].

Moreover, while the differences indeed appear small when judged by the median statistic, these differences are occasionally very large and positive for individual neural sites when the distribution of these differences are examined (up to 0.4 increase in predictivity on OOD). We included the histogram of improvements across individual sites for 5 example models in the new supplementary figure S7, providing additional support for our preference for neuron-layer alignment over the area-layer approach and updated the main text on lines [224-227]. The following figure shows the histogram of differences between the two approaches for 2 example models showing occasionally large improvement in neural predictivity on both ID and OOD when using the layer-neuron approach despite almost no change in the mean (dashed vertical line).

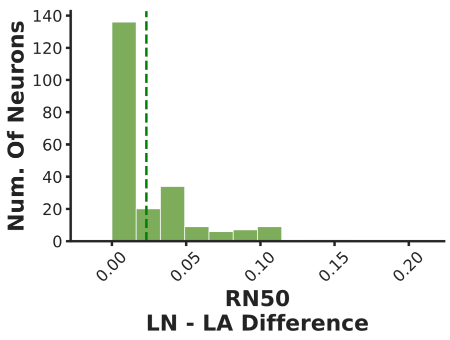

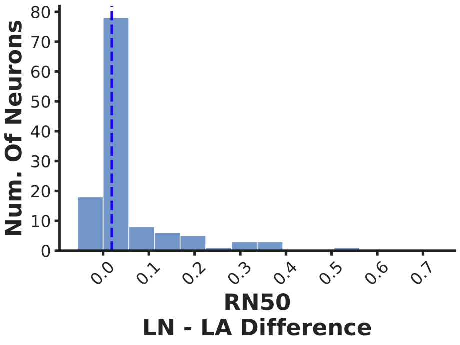

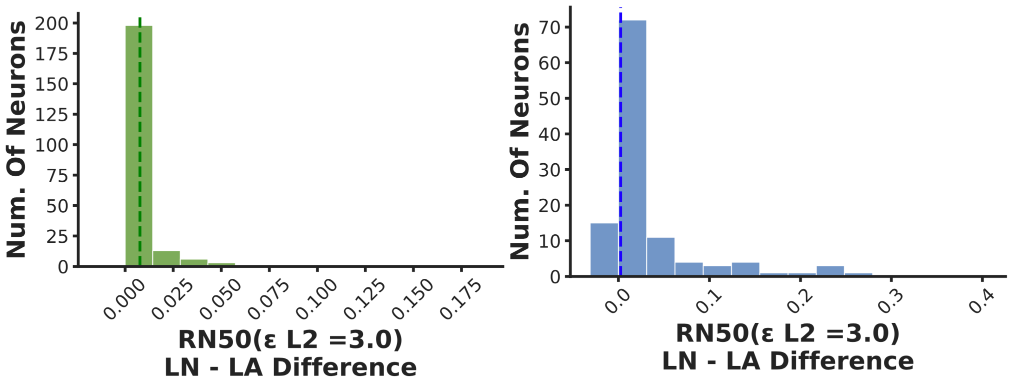

"Fig 4b: the bars are in a different order than the legend. Please order the legend the same as the bars to avoid confusion. Also in the text the order in which the alterations are explained is switched between ImageNet Adversarial and ImageNet Rendition, adjusting the order to match Fig 4a might be good."
We thank the reviewer for bringing this inconsistency to our attention. We updated the figure legend accordingly.

"Fig 5c: you omitt two of the values from 5b, the 4/255 and 8/255, why?"
We did not include these two models (4/255 and 8/255) in figure 5c because they were trained on an attack with a different norm (L_{infinity}), different number of attack steps and step sizes compared to the rest of the models. We revised the description of Fig 5c to clarify this distinction and our logic for not including these models.

Reviewer #2

"I think the overall structure of the article feels somewhat disjointed. It would be more reader-friendly if each section were organized around a clear theme, specifically outlining why that section is essential to answering the primary question."
We appreciate the comment about readability of our manuscript. To address the reviewer's comment, we made several changes to the text to improve readability and to further clarify the main point in each subsection and how they are related to each other. Specifically, we added more context in the beginning of each of the subsections of the Results section to further clarify the goal of experiments under each subsection. We also added a paragraph at the beginning of the results section that summarizes all of the sections in the results section with a brief overview of the main message discussed in each of them. We believe that these changes have substantially improved the readability of our manuscript. You can view these changes on lines [97 - 109], [177 - 178], [197 - 198], [336 - 341].

"Monkey S's data exhibit similar predictivity to both natural and synthetic stimuli in both sessions (Fig 2c). It would be insightful to discuss how this monkey's data differs from others."
We thank the reviewer for bringing this difference to our attention. We note two differences between monkey S and the other two monkeys in our dataset that could potentially explain the observed differences in predictivity in this monkey: 1) the receptive field of neurons in

monkey S are more peripherally located compared to other monkeys. We noticed these differences from the classic receptive field maps in Bashivan et al. 2019 and also confirmed them by examining the synthetically generated images for each of the monkeys which were more peripheral for this monkey compared to other ones; 2) we observed that many of the synthetically generated images for Monkey S covered a smaller part of the images compared to those that were generated for monkey M. This difference could have been due to the specifics of the linear mapping model that was learned during the prediction procedure. The images covering larger parts of the field could have potentially led to invoking more complex population responses that were more difficult to be captured by the DNN models tested in this work. We included a summary of this description in on lines [156-162].

"Section 2.2 contrasts Layer-Area mapping with Layer-Neuron mapping. As indicated by Fig 3a and 3d, the disparity isn't significant. I feel this section doesn't critically support the main argument. It might be more appropriate to directly use the layer-neuron analysis and relegate the comparative discussion to supplementary materials."

We thank the reviewer for their comment. We would like to note that the results in Figure 3 and S3 both support our argument in section 2.2 for preferring the neuron-layer approach over area-layer. The data from all animals and all sessions confirms that neurons recorded from the same area are best mapped to two or more layers in the neural network model (Fig. 3a and b). While we agree with the reviewer that across all models, the difference between the two methods seems to be small, we note that even though the in-distribution (ID) improvements were small, they were statistically significant with $p<0.0001$. Moreover, while the differences between the two approaches appear small when judged by the median statistic, these differences are occasionally very large and positive for individual neural sites (up to 0.4 increase in predictivity on OOD). This is evident when the distribution of these differences are examined for individual models. We included the histogram of improvements across individual sites for 5 example models in the new supplementary Figure S7, providing additional support for our preference for neuron-layer alignment over the area-layer approach. The following histograms are for 2 example models showing occasionally large improvements in both ID and OOD neural predictivity for a subset of neuronal sites at almost no cost for other neurons when using the layer-neuron approach. We update the text on lines [224-227] to highlight this point.

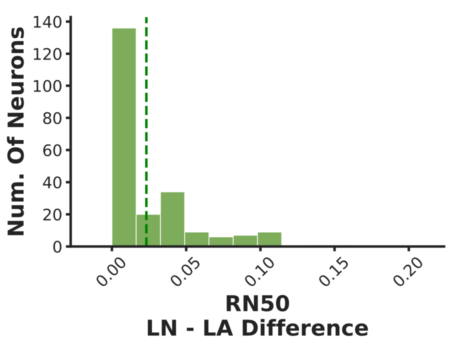

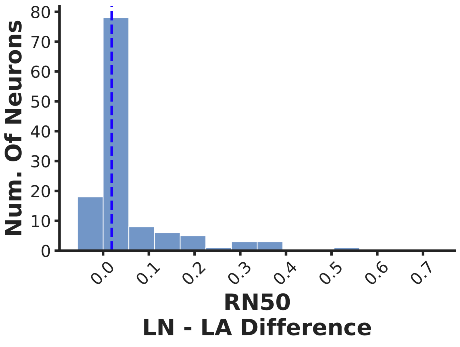

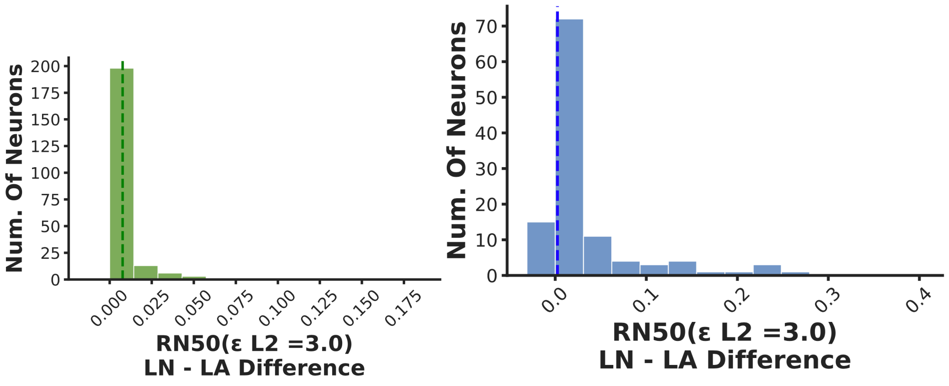

"The title of section 2.3 might be more aptly named "Is Recognition Generalization Ability a Reliable Indicator of Neural Predictivity?"".

We thank the reviewer for their suggestion. We revised the title of this section accordingly.

"I have a question about the predictivity measure (lines 552-558). Since the authors utilized PCs of layer activations to reduce dimensionality, were the PCs estimated from natural images also used to train the predictive model on synthetic data? If this is the case, isn't it unfair for predicting OOD responses? Many pertinent features might exist in the original model layer but might be omitted due to the projection to PCs from another domain. I'm curious why the authors didn't simply regress the raw model features to neural responses. Considering it's merely a linear mapping, I assume it would still be feasible."

We understand the reviewer's concern about the potential effect of applying naturally-tuned PCA-transformation on predicting responses to synthetically generated ones. To address the reviewer's concern, we repeated the experiments in Fig 2e but this time without the use of PCA and by directly fitting the PLS regression model on the model activations from ResNet50. The results from this analysis reproduced almost identical ones to those in which we used PCA and PLS together. This suggests that the naturally-tuned PCA is not a confounding factor in predicting the responses to synthetic images. We added the result of this analysis as a new supplementary figure S8 and added additional text in section 4.2 lines [600-604].

"There are numerous error bars in the bar plots, like in Fig 2c, 2d, Fig 5b, Fig 6, etc. How are these defined? Are they standard deviations or standard errors of the mean? Furthermore, over which variables are these calculations made?"

All error bars were calculated as the variance across 5 repetitions of each analysis. We add this detail to Figures 2, 5, 6, and 7 captions.

"The bibliography section needs revision; numerous references lack titles."

We thank the reviewer for bringing this issue to our attention. We updated the references and made sure each reference contains all the information.

"In Fig 3, incorporating a legend for "green" and "blue" would enhance clarity."

We thank the reviewer for their suggestion, we have added the legend to Fig 3a accordingly.

?On line 146, "Fig 1e" should be corrected to "Fig 2e".
Captions for Fig 2d and 2e appear to be absent."
We thank the reviewer for bringing this error to our attention. We updated the manuscript and the caption and added the missing information.

"A comprehensive list of models employed in the analysis should be included. For instance, line 113 mentions "40 artificial neural networks", and line 216 refers to "33 neural network models". Which are these models? Were they developed by the authors, or sourced from prior studies? If the former, will they be made publicly available?"
We double checked the total number of models used in our analysis and the correct number is 38. All 38 models are now listed in section 4.5 (line [681 - 690]). The 33 models referred to in the text includes all of the original models except 5 models that lacked the linear classification layers. These models consist of InsDis RN50, Local Aggregation RN50, Place365 RN50 and two MoCo models with different architectures trained by ourselves. We revised the text on lines [245-247] to clarify this point.
